# The Influence of Personality Traits on Postpartum Depression: A Systematic Review Based on the NEO-FFI Scale

**DOI:** 10.3390/diseases12050082

**Published:** 2024-04-25

**Authors:** Oana Neda-Stepan, Cătălina Giurgi-Oncu, Andreea Sălcudean, Elena Bernad, Brenda-Cristiana Bernad, Virgil Radu Enătescu

**Affiliations:** 1 Doctoral School, “Victor Babes” University of Medicine and Pharmacy, Eftimie Murgu Square 2, 300041 Timisoara, Romania; oana.neda-stepan@umft.ro (O.N.-S.); bernad.brenda@umft.ro (B.-C.B.); 2 Department VIII—Neurosciences, Discipline of Psychiatry, “Victor Babes” University of Medicine and Pharmacy, Eftimie Murgu Square 2, 300041 Timisoara, Romania; catalina.giurgi@umft.ro (C.G.-O.); enatescu.virgil@umft.ro (V.R.E.); 3 Discipline of Sociobiology, Department of Ethics and Social Sciences, George Emil Palade University of Medicine, Pharmacy, Science and Technology of Targu Mures, 540136 Targu Mures, Romania; 4 Department of Obstetrics and Gynecology, “Victor Babes” University of Medicine and Pharmacy, Eftimie Murgu Square 2, 300041 Timisoara, Romania; bernad.elena@umft.ro

**Keywords:** postpartum depression, personality inventory, psychological stress, maternal health

## Abstract

Postpartum depression (PPD) is a significant global health concern with profound implications for mothers, families, and societies. This systematic review aims to synthesize current research findings to understand better how personality traits, as assessed by the NEO Five-Factor Inventory (NEO-FFI), contribute to the development and progression of PPD. Conducted in January 2024, this review searched major databases like PubMed, PsycINFO, and Scopus up to December 2023, focusing on the NEO-FFI’s role in evaluating PPD. Following PRISMA guidelines, studies were selected based on strict criteria, including the exclusive use of NEO-FFI for personality assessment and a focus on postpartum women. A total of seven studies were included after a rigorous two-step screening process, and their data were qualitatively synthesized. The review covered a total of 4172 participants, with a prevalence of clinically significant postpartum depression symptoms ranging from 10.6% to 51.7%. Notably, Neuroticism emerged as a significant predictor of PPD, with odds ratios ranging from 1.07 (95% CI: 0.96–1.20) in some studies to as high as 1.87 (95% CI: 1.53–2.27) in others. In contrast, traits like Extraversion and Conscientiousness generally showed protective effects, with lower scores associated with reduced PPD risk. For instance, Extraversion scores correlated negatively with PPD risk (Beta = −0.171) in one study. However, the impact of other traits such as Openness and Agreeableness on PPD risk was less clear, with some studies indicating negligible effects. The review highlights Neuroticism as a consistent and significant predictor of PPD risk, with varying impacts from other personality traits. The findings suggest potential pathways for targeted interventions in maternal mental health care, emphasizing the need for comprehensive personality evaluations in prenatal and postnatal settings.

## 1. Introduction

Postpartum depression (PPD) represents a significant mental health concern, affecting a considerable proportion of new mothers worldwide [1,2]. Characterized by persistent feelings of sadness, anxiety, and fatigue, PPD can severely impact maternal well-being and child development [3,4,5]. Emerging evidence suggests that individual personality traits, as measured by the NEO Five-Factor Inventory (NEO-FFI), might play a critical role in predisposing certain women to PPD [6,7,8]. PPD can be influenced by a variety of factors, including biological, psychological, and social elements. Hormonal changes, genetic predispositions, life stressors, and social support systems are among the diverse influences that can impact the likelihood and severity of PPD [2,7,8]. Within this complex interplay of factors, personality traits emerge as a significant psychological dimension that warrants closer examination. These individual differences in traits, how people typically think, feel, and behave, may not only help explain the variance in PPD experiences but also offer predictive value for identifying those at greater risk.

Personality traits, as conceptualized by the NEO-FFI, encompass five major domains: Neuroticism, Extraversion, Openness, Agreeableness, and Conscientiousness [9]. Each of these traits has been individually linked to various mental health outcomes. For instance, high levels of neuroticism have been associated with a greater risk of developing mood and anxiety disorders [10], while traits like extraversion and conscientiousness often correlate with better psychological resilience [11,12]. Given these links, it is plausible that these personality dimensions, alone or in association with other significant factors, could significantly affect the vulnerability and resilience factors in the context of PPD and may even influence pregnancy outcomes [13,14,15,16,17].

Recent studies have begun to explore the relationship between these personality traits and postpartum mental health [18,19]. For example, women scoring higher in neuroticism have been reported to experience more severe symptoms of PPD [20]. Conversely, traits such as agreeableness and conscientiousness might offer a protective effect. However, the extent and nature of these associations remain unclear, necessitating a comprehensive review of the existing literature, moreover in the context of a multitude of instruments being created to determine personality traits.

Furthermore, understanding the role of personality traits in PPD and depressive symptoms after birth is crucial for improving preventative strategies and treatment approaches [21]. Identifying at-risk individuals based on their personality profiles could enable healthcare providers to tailor interventions more effectively, thereby reducing the incidence and severity of PPD.

Therefore, this review aims to synthesize the current research findings, providing a clearer understanding of how personality traits, as assessed by the NEO-FFI, contribute to the development and progression of PPD. This is particularly vital in the context of global health, where PPD remains a prevalent yet often under-addressed issue, with significant implications for mothers, families, and societies at large. Addressing only the NEO-FFI scale in this systematic review is relevant due to the different nature of psychological domains and traits that different tools assess, but that do not correspond altogether.

## 2. Materials and Methods

### 2.1. Eligibility Criteria

The inclusion criteria for this review were as follows: (1) studies that examined the relationship between NEO-FFI-assessed personality traits and postpartum depression; (2) research that included populations of postpartum women assessed using the NEO-FFI; (3) studies providing clear methodologies for assessing both personality traits and postpartum depression; (4) research published in peer-reviewed journals in English.

The exclusion criteria were as follows: (1) studies not specifically using the NEO-FFI for personality assessment; (2) research focusing on perinatal depression not classified as postpartum depression; (3) non-peer-reviewed articles, in vitro studies, conference proceedings, general reviews, systematic reviews, meta-analyses, case reports, commentaries, and editorial letters; (4) research published after 2010, to avoid changes in definitions of psychologic features and psychiatric diagnosis.

### 2.2. Information Sources

This systematic review, conducted in January 2024, was aimed at assessing the role of personality traits in postpartum depression, with a specific focus on assessments made using the NEO Five-Factor Inventory (NEO-FFI). A comprehensive search was performed across major electronic databases, including PubMed, PsycINFO, and Scopus, with the literature scope extending up to December 2023.

### 2.3. Search Strategy

Our search strategy was designed to capture a broad spectrum of studies related to personality traits and postpartum depression, using an expanded set of keywords such as “Postpartum Depression”, “Personality Traits”, “NEO-FFI”, “Maternal Mental Health”, “Psychological Assessment”, “Big Five Personality Factors”, “Neuroticism and Postpartum”, “Extraversion in Motherhood”, “Postnatal Psychological Well-being”, “Agreeableness and Maternal Health”, “Conscientiousness in Postpartum Women”, and “Emotional Stability in New Mothers”.

The search strings employed were combinations of these terms, such as: (“Postpartum Depression” OR “PPD”) AND (“Personality Traits” OR “NEO Five-Factor Inventory” OR “NEO-FFI” OR “Big Five Personality Factors”) AND (“Maternal Mental Health” OR “Psychological Assessment” OR “Postnatal Psychological Well-being” OR “Emotional Stability in New Mothers”). This approach aimed to ensure a thorough and inclusive retrieval of relevant literature, capturing diverse studies that explore the intricate relationship between various personality dimensions and postpartum depression.

The review adhered to the Preferred Reporting Items for Systematic Reviews and Meta-Analyses (PRISMA) guidelines [22], ensuring a structured and transparent approach. The review protocol was registered on the International Prospective Register of Systematic Reviews (PROSPERO) [23]. The systematic review was registered on the Open Science Framework database, with the registration code osf.io/r467d.

### 2.4. Selection Process

A two-step screening process was employed. Initially, titles and abstracts were screened to exclude irrelevant studies. The full texts of the remaining articles were then reviewed to assess their eligibility based on the predefined criteria. This process was conducted independently by two researchers, with any discrepancies resolved through discussion or consultation with a third researcher.

### 2.5. Data Selection Process and Quality Assessment

**Figure 1:**
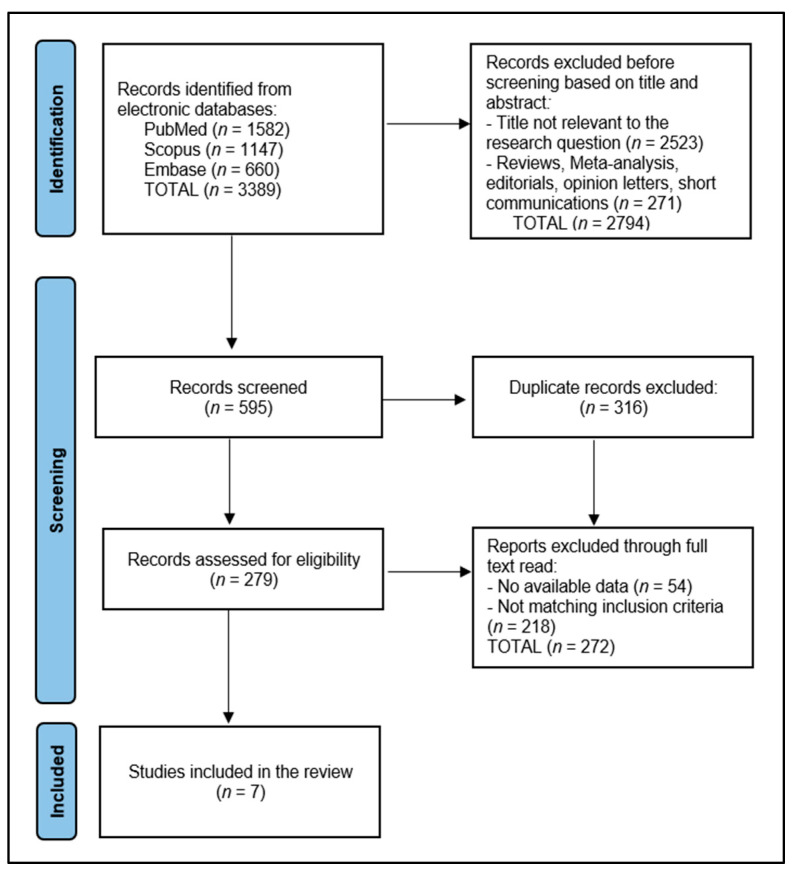
PRISMA flowchart.

Data from the included studies were extracted and included authors, year of publication, study design, sample size, main findings, and any relevant notes on the assessment tools used (specifically the NEO-FFI) [24]. Quality assessment of the studies was performed using the Newcastle–Ottawa Scale for cohort and case-control studies and the Joanna Briggs Institute (JBI) checklist for cross-sectional studies. This assessment helped evaluate the risk of bias and the methodological quality of the included studies. A total of 7 studies were included in the systematic review, according to the PRISMA flowchart presented in Figure 1. Data in the current study were extracted and classified into four sections: (1) study characteristics; (2) patients’ characteristics; (3) pregnancy characteristics; (4) NEO-FFI results.

### 2.6. Data Items

In this systematic review, the collected data encompassed a broad array of variables, thoroughly delineating the landscape of existing literature on postpartum depression and personality traits. Key data items included authorship and publication details, geographic and temporal contexts of the studies, and methodological approaches such as study design and quality assessments. These elements provided foundational insights into the diversity and scientific rigor of the research corpus. Additionally, participant demographics like age, psychiatric assessments, and socio-economic factors were meticulously recorded, alongside critical psychological evaluations at various postpartum stages using standardized tools such as the Edinburgh Postnatal Depression Scale. This comprehensive data collection strategy, rooted in the PRISMA guidelines, ensured a robust synthesis of findings, facilitating a nuanced understanding of how specific personality traits might influence postpartum depression outcomes.

## 3. Results

### 3.1. Study Characteristics

The systematic review included seven studies, as detailed in Table 1 [25,26,27,28,29,30,31]. These studies were conducted in a range of countries, including Croatia, Poland, Spain, China, Iran, and Romania. The review covered research published between 2012 and 2020, highlighting a recent focus on this area.

The studies employed various research designs: four were cross-sectional (Maliszewska et al. [26,28], Tian et al. [29], and Roman et al. [31]), two were prospective cohorts (Imsiragic et al. [25] and Marin-Morales et al. [27]), and one was a case-control study (Tian et al. [29]). This mix of designs provided a comprehensive view of the relationship between personality traits and postpartum depression.

In terms of quality assessment, the majority of studies were rated as having medium quality, indicating a reasonable level of methodological rigor. However, two studies (Maliszewska et al. [28] and Farzaneh et al. [30]) were rated as low quality, suggesting potential limitations in their findings or methodologies.

### 3.2. Patients’ Characteristics

The total patient count across the studies was 4172, varying from 116 in Marin-Morales et al. [27] to 1785 in Tian et al. [29]. The age of participants generally centered around the early thirties, with an average age ranging from 29.33 years in Roman et al. [31] to 31.31 years in Marin-Morales et al. [27].

Regarding psychiatric/psychological assessments, the studies utilized various tools to evaluate postpartum depression and related symptoms. The prevalence of clinically significant scores on the Edinburgh Postnatal Depression Scale (EPDS) varied widely, from 10.6% in the study by Maliszewska et al. [28] to 51.7% in the study by Imsiragic et al. [25]. Other assessments included the Impact of Events Scale-Revised (IES-R) and the Patient Health Questionnaire-9 (PHQ-9), with significant findings of postnatal anxiety and depression symptoms across the different cohorts.

The timing of assessments varied across studies, ranging from 3–5 days to 6–9 weeks after delivery in the study by Imsiragic et al. [25], to assessments conducted in the first trimester and four months after delivery by Marin-Morales et al. [27], and up to six months post-delivery in the Farzaneh et al. study [30].

Other characteristics revealed a wide range of demographic and socioeconomic factors. For instance, a notable percentage of participants had secondary education or less, with 51.1% reported in the study by Imsiragic et al. [25]. Employment status also varied, with 24.1% unemployment in the same study. In contrast, Maliszewska et al. [28] reported that 80% of their cohort had higher education, and 88% were employed, as presented in Table 2.

### 3.3. Pregnancy Characteristics

In examining the characteristics and outcomes of pregnancy as reported in Table 3, a comparative analysis across several studies reveals key trends and variations in mode of delivery, breastfeeding practices, smoking habits, and other complications associated with childbirth. The mode of delivery across the studies shows a predominance of vaginal deliveries, with percentages ranging from 46.1% in Roman et al. [31] to 78.2% in Imsiragic et al. [25]. This variability suggests differences in medical practices, patient preferences, or clinical indications for C-sections across the study populations. Notably, Roman et al. [31] reported the highest rate of C-sections (53.9%), indicating a significant departure from other studies and possibly reflecting specific population characteristics or clinical guidelines influencing delivery mode decisions.

Breastfeeding rates were generally high where reported, with Imsiragic et al. [25] documenting an 83.3% rate and Maliszewska et al. [26] observing a slight increase from 82.2% in the first week to 86.3% in the fourth week. However, the variation in reporting times and the absence of data from several studies (Marin-Morales et al. [27], Tian et al. [29], Farzaneh et al. [30], and Roman et al. [31]) limit comprehensive cross-study comparisons.

Smoking rates were explicitly reported only in the studies by Maliszewska et al. [26] and [28], with relatively low prevalence rates of 9.54% and 9.26%, respectively. Also, the studies varied widely in the type and frequency of reported complications. Emergency C-section rates, preterm deliveries, and complications during or after labor were notably diverse. For instance, Imsiragic et al. [25] highlighted complications during or after labor (28.8%) and emergency C-sections (10.2%), while Maliszewska et al. [28] reported a 10.2% rate of preterm delivery. Marin-Morales et al. [27] provided specific data on neonatal outcomes with a low APGAR score occurrence (1.7%). Interestingly, Farzaneh et al. [30] diverged from the pattern by focusing on the relationship between postpartum depression and social support, identifying a significant negative correlation. This finding underscores the importance of considering psychological and social factors alongside physical health metrics in understanding postpartum well-being.

### 3.4. NEO-FFI Results

The analysis of Table 4 provides objective data on the association between personality traits measured by the NEO-FFI scale and the risk of postpartum depression. The results, derived from various studies, reveal significant insights into how specific personality traits influence the likelihood of developing postpartum depression. Imsiragic et al. [25] found a marginally increased risk of postpartum depression associated with higher levels of Neuroticism (Odds Ratio [OR] = 1.07, 95% Confidence Interval [CI]: 0.96–1.20). Other personality traits such as Extraversion (OR = 0.95, 95% CI: 0.88–1.03), Conscientiousness (OR = 0.97, 95% CI: 0.90–1.04), Openness (OR = 0.98, 95% CI: 0.93–1.04), and Agreeableness (OR = 1.04, 95% CI: 0.97–1.12) showed negligible to no significant effects on the risk of developing postpartum depression.

Maliszewska et al. [26] reported more pronounced effects, with Neuroticism significantly increasing the risk of depression (OR = 1.87, 95% CI: 1.53–2.27). Lower scores in Extraversion (OR = 0.69, 95% CI: 0.57–0.82), Conscientiousness (OR = 0.79, 95% CI: 0.67–0.92), Openness (OR = 0.84, 95% CI: 0.72–0.99), and Agreeableness (OR = 0.87, 95% CI: 0.75–1.01) were associated with a reduced risk, indicating protective effects against postpartum depression.

In a subsequent study, Maliszewska et al. [28] found that Neuroticism remained a significant predictor of depression risk (OR = 1.64, 95% CI: 1.34–2.01), with reduced scores in Extraversion (OR = 0.76, 95% CI: 0.58–0.87) and Conscientiousness (OR = 0.78, 95% CI: 0.66–0.93) again associated with lower risk levels.

Tian et al. [29] focused exclusively on Neuroticism, observing a modest increase in depression risk (OR = 1.12, 95% CI: 1.09–1.21), without addressing the impact of other personality traits. Moreover, Farzaneh et al. [30] provided a nuanced view with beta coefficients, indicating a positive correlation of Neuroticism with depression risk (Beta = 0.368) and negative correlations for Extraversion (Beta = −0.171) and Conscientiousness (Beta = −0.162). These findings suggest that higher levels of Neuroticism may increase the risk of postpartum depression, while higher levels of Extraversion and Conscientiousness could potentially reduce it.

Roman et al. [31] presented data as standardized total effects, showing minimal associations across all personality traits with depression risk, suggesting no substantial impact on postpartum depression risk in their analysis. Specifically, the standardized total effects for Neuroticism (0.01, 95% CI: 0.03–0.10), Extraversion (−0.03, 95% CI: 0.02–0.04), Conscientiousness (−0.05, 95% CI: −0.06–0.007), Openness (−0.01, 95% CI: 0.02–0.005), and Agreeableness (−0.02, 95% CI: −0.02–0.06) were found to be very small, indicating negligible effects on depression risk.

## 4. Discussion

This systematic review provides critical insights into the complex relationship between personality traits and the risk of postpartum depression. Patient characteristics across the studies reveal significant diversity in demographic and socioeconomic backgrounds. The variation in age, education, employment status, and psychiatric assessments such as the Edinburgh Postnatal Depression Scale (EPDS) scores underscores the complexity of PPD and its multifactorial nature. The wide range in the prevalence of clinically significant EPDS scores, from 10.6% to 51.7%, reflects this complexity and indicates that PPD is a significant health concern across different populations. The timing of assessments also varies, which could influence the reported prevalence rates and should be considered when interpreting the results.

The variation in population characteristics across the included studies, spanning age, education, employment status, and cultural backgrounds, provides a rich tapestry for understanding the nuanced relationships between personality traits and PPD. Therefore, higher education levels and employment might correlate with better access to resources and support systems, potentially moderating the impact of certain personality traits on PPD risk. Conversely, demographic factors such as younger age or lower socioeconomic status could exacerbate the influence of traits like neuroticism on PPD. Such diversity necessitates a careful consideration of context when interpreting the relationships between personality traits and PPD, underscoring the importance of a multifaceted approach that accounts for the complex interplay of individual, socio-economic, and cultural factors in shaping mental health outcomes postpartum.

Pregnancy characteristics, such as mode of delivery, breastfeeding practices, smoking habits, and other complications, show notable variations across the studies. The predominance of vaginal deliveries in some studies contrasted with higher C-section rates in others like Roman et al. [31], indicating potential cultural or medical practice differences. The generally high breastfeeding rates are encouraging, but the variability in reporting and absence of data in some studies limit comprehensive understanding. The diversity in reported complications and the significant negative correlation between postpartum depression and social support found in Farzaneh et al. [30] highlight the importance of considering both physical and psychosocial factors in understanding PPD.

The review’s core findings regarding the NEO-FFI scale’s role in assessing PPD risk are particularly enlightening. The variation in the impact of personality traits like Neuroticism, Extraversion, and Conscientiousness on PPD risk across different studies suggests that personality traits may play a significant but complex role in PPD development. For instance, the pronounced effects of Neuroticism in increasing depression risk, as reported by Maliszewska et al. [26], contrast with minimal associations found in Roman et al. [31]. This disparity might be due to differences in study designs, populations, and methodologies. The consistent finding across several studies that higher levels of Neuroticism may increase PPD risk, while traits like Extraversion and Conscientiousness might offer protective effects, provides valuable insights for future research and clinical practice [32,33,34].

Building upon the findings from the systematic review by Puyane et al. [35], the association between neuroticism and postpartum depression is further elucidated. This study, included in their meta-analysis, reinforces the notion that certain personality traits, particularly neuroticism, significantly contribute to the risk of developing PPD. The quantifiable association, with an odds ratio of 1.37 (1.22–1.53), underscores the robustness of this relationship. The study’s emphasis on neuroticism aligns with existing literature suggesting that individuals with higher levels of neuroticism are more prone to experience negative emotional states, which could predispose them to PPD. By identifying women with high levels of neuroticism, healthcare professionals can offer targeted support and monitoring, potentially mitigating the onset or severity of PPD [35,36,37]. Additionally, the study’s findings about vulnerable personality style and trait anxiety as contributing factors to PPD offer a broader perspective on the psychological risk factors involved.

In the intricate exploration of personality changes and their implications on perinatal depressive symptoms, studies by Leikas et al. [38] and Serra et al. [39] offer valuable insights through their specific findings and methodologies. Leikas et al. observed nuanced shifts in neuroticism and extraversion facets, noting decreases in excitability and affective facets of neuroticism, while impulsivity and self-consciousness facets increased postpartum. These changes were further influenced by the mother’s perception of her child’s temperament. Serra et al. elucidated the direct correlation between neuroticism, couple conflict, and depressive symptoms, highlighting neuroticism’s mediating role in linking family psychiatric history with perinatal depression. However, despite their contributions, these studies were not included in the final assessment of our systematic review due to methodological divergences in personality assessment tools: Leikas et al. utilized the NEO-PI-R survey, while Serra employed the BIG-5 questionnaire.

Similarly, other studies focus on a more prevalent issue than PPD, represented by depressive symptomatology after labor. The study conducted by van Bussel et al. offers a unique perspective on the role of maternal orientations and their influence on depressive symptoms during and after pregnancy [40]. This longitudinal study, involving 403 pregnant women, utilized the EPDS and HADS-D scales across different stages of pregnancy and the postpartum period. The findings suggest a significant association between the ‘Regulator’ orientation and increased depressive symptoms, both during pregnancy and in the postpartum period. This orientation, characterized perhaps by a desire for control and regulation in the maternal experience, may contribute to heightened stress or anxiety, thereby increasing vulnerability to depressive symptoms. Conversely, the ‘Facilitator’ orientation, which might be indicative of a more adaptive and flexible approach to motherhood, showed a negative association with depressive symptoms, although this was not consistently significant across all points of measurement.

The research by Spry et al. [41] provides significant insights into how parental personality traits, assessed before conception, can impact family dynamics, the development of depressive symptoms, and child development during the critical first thousand days of life. This study demonstrates that parental traits such as agreeableness, conscientiousness, emotional stability, extraversion, and openness are not just critical for the parents’ own mental health and parenting style, but also profoundly influence the household’s social and financial context, as well as the temperamental characteristics of their offspring. These findings suggest that understanding and supporting parental personality aspects well before the conception of a child could lead to more effective strategies for fostering positive family environments and child developmental outcomes.

The exclusive post-delivery timing of personality assessments highlighted in our review suggests a compelling direction for future research: investigating personality trait variations across the perinatal period. Understanding how personality traits may shift from before to after childbirth could profoundly impact our comprehension of their relationship with postpartum depression (PPD). This line of inquiry promises to unravel the complex interplay between evolving personality dynamics and PPD, offering potential for novel preventive and therapeutic approaches that consider the temporal changes in personality traits during this critical life transition.

The heuristic value of the NEO-FFI across different health scenarios shows evidence that the Five Factor Model personality traits exert significant and varied influences on both physical and psychological health outcomes [8]. Neuroticism, often linked with heightened vulnerability to mental health challenges such as anxiety and depression, has also been associated with increased risk for physical health issues, including heart disease and chronic pain, underscoring its pervasive impact on overall health. In contrast, extraversion, characterized by its protective qualities, has been shown to foster better mental health, enhanced social support, and potentially improved immune system functioning. The trait of openness, with its association with cognitive flexibility and creativity, suggests a proactive approach to health management and a lower risk of cognitive decline. Agreeableness, through its facilitation of social harmony and stress reduction, contributes positively to managing and adjusting to chronic health conditions. Lastly, conscientiousness is linked to healthier lifestyle choices, lower substance abuse rates, and proactive engagement in preventive health measures, highlighting its role in promoting longevity and reducing morbidity. This approach not only reinforces the instrument’s broad applicative value but also underscores the potential of personality assessments in enhancing our understanding of health-related vulnerabilities and strengths.

This systematic review, while providing valuable insights into the relationship between NEO-FFI-assessed personality traits and postpartum depression (PPD), has several limitations. The exclusive reliance on the NEO-FFI scale, although ensuring methodological coherence, restricts the breadth of personality aspects studied and may not capture nuances revealed by other personality assessment tools. The heterogeneity of the included studies, encompassing cross-sectional, prospective cohorts, and a case-control study, introduces variability in outcomes, potentially affecting the consistency and comparability of findings. The diversity and discrepancies in findings, notably regarding traits like neuroticism, underscore the complexity of psychological research and the necessity of cautious interpretation of these results. Furthermore, the varying quality of the studies, with some rated as medium and others as low, raises concerns about the reliability and generalizability of the results. The decision to conduct a narrative synthesis, rather than a meta-analysis, due to the expected heterogeneity, while understandable, limits the ability to quantitatively assess the strength of associations between personality traits and PPD.

While our systematic review included a small number of seven studies, this was a result of our rigorous adherence to a strict set of inclusion criteria focusing on the NEO-FFI scale and postpartum depression. We prioritized methodological consistency and the specificity of assessment tools, which limited the scope of available research but enhanced the precision and relevance of our analysis. This approach, while restrictive, was necessary to maintain the scientific integrity.

## 5. Conclusions

This systematic review conclusively demonstrates that personality traits, specifically as measured by the NEO-FFI, have a significant impact on the risk and progression of PPD. Our findings underscore Neuroticism as a robust predictor of PPD, indicating a pressing need for its consideration in prenatal and postnatal healthcare strategies. The protective effects of traits like Extraversion and Conscientiousness against PPD further emphasize the role of comprehensive personality assessments in maternal mental health. These results not only augment our understanding of the psychological factors contributing to PPD but also pave the way for more tailored and effective interventions. By integrating personality assessments into routine maternal care, healthcare providers can better identify and support women at higher risk of PPD, potentially mitigating its impact on mothers and families. Future research should aim to expand upon these findings, exploring the intricate dynamics between various personality traits and PPD in diverse populations and settings, thereby enhancing our capability to address this global health concern more effectively.

## Figures and Tables

**Table 1:** Characteristics of the reviewed studies.

First Author	Country	Year of Publication	Study Design	Quality Assessment
Imsiragic et al. [25]	Croatia	2014	Prospective cohort	Medium
Maliszewska et al. [26]	Poland	2017	Cross-sectional	Low
Marin-Morales et al. [27]	Spain	2014	Prospective cohort	Medium
Maliszewska et al. [28]	Poland	2017	Cross-sectional	Medium
Tian et al. [29]	China	2012	Case-control	Medium
Farzaneh et al. [30]	Iran	2020	Cross-sectional	Low
Roman et al. [31]	Romania	2019	Cross-sectional	Medium

**Table 2:** Patients’ characteristics in the reviewed studies.

First Author	Number of Patients	Age	Psychiatric/Psychological Assessments	Time of Assessment	Other Characteristics
Imsiragic et al. [25]	262	30 years (median)	Constant fear of labor outcome: 38.9%; EPDS score: 51.7% clinically significant; IES-R score: 45.6% clinically significant	3–5 days and 6–9 weeks after delivery	51.1% had secondary school or less; 24.1% were unemployed.
Maliszewska et al. [26]	387	30.37 years (average)	Positive psychiatric history: 7.5%; positive family history: 4.4%; EPDS score: 28.9% clinically significant	4–8 weeks after delivery	79.1% had higher education; 9.9% were unemployed; 81.1% were married and primiparous (56.9%).
Marin-Morales et al. [27]	116	31.31 years (average)	EPDS score: 19.2% clinically significant; SCL-90 mean score: 0.73	1st trimester and 4 months after delivery	82.8% planned pregnancies; 50.5% multiparous; 27.5% had primary education, 45.4% secondary, and 27.1% university; 65.1% were employed at the time of pregnancy.
Maliszewska et al. [28]	548	30.19 years (average)	EPDS score: 10.6% clinically significant; PHQ-9 score: 13.3% clinically significant; 6.25% had a history of psychiatric disorder	1st week after delivery, 4 weeks, and 3 months after delivery	80% had higher education; 88% were employed and married (80.1%); 54.2% were primiparas.
Tian et al. [29]	1785	NR	NR	NR	Postpartum depression risk (OR): age 0.95(0.03–0.06); premenstrual symptoms 1.11(1.07–1.15); education 0.88(0.83–0.93); occupation 0.93(0.88–0.98); lifetime stressful events 1.23(1.15–132); childhood sexual abuse 1.80(1.27–2.54).
Farzaneh et al. [30]	200	17–41 (range)	EPDS score: 24.5% clinically significant for depression	6 weeks to 6 months after delivery	47.5% had a body mass index (BMI) of less than 18.5 (underweight), 5.5% had more than one child, 81.5% were housewives, and 69.5% had a diploma or higher education.
Roman et al. [31]	672	29.33 years (average)	24.3% had postnatal anxiety	3–4 days after birth and 2 weeks after delivery	63.8% lived in urban areas; 40.9% had a college degree or higher; 83% were married

NR—Not Reported; EPDS—Edinburgh Postnatal Depression Scale; IES-R—Impact of Events Scale (revised); SCL—Symptom Checklist; PHQ—Patient Health Questionnaire; OR—Odds Ratio.

**Table 3:** Pregnancy characteristics and outcomes.

First Author	Mode of Delivery	Breastfeeding	Smoking	Other Complications
Imsiragic et al. [25]	78.2% vaginal	83.3%	NR	Emergency C-section: 10.2%; complications during or after labor: 28.8%
Maliszewska et al. [26]	69.8% vaginal	82.2% in the first week, 86.3% in the fourth week	9.54%	C-section: 30.2%
Marin-Morales et al. [27]	51.7% vaginal	NR	NR	APGAR score < 7: 1.7%
Maliszewska et al. [28]	68.7% vaginal	65.7%	9.26%	10.2% preterm delivery; 24.2% hospitalized
Tian et al. [29]	NR	NR	NR	NR
Farzaneh et al. [30]	NR	NR	NR	There was a significant negative relationship between postpartum depression and social support (r = −0.027, *p* < 0.01).
Roman et al. [31]	46.1% vaginal	NR	NR	C-section: 53.9%

NR—Not Reported.

**Table 4:** NEO-FFI results in assessment of depression risk based on personality type.

First Author	Neuroticism	Extraversion	Conscientiousness	Openness	Agreeableness
Imsiragic et al. [25]	1.07 (0.96–1.20)	0.95 (0.88–1.03)	0.97 (0.90–1.04)	0.98 (0.93–1–04)	1.04 (0.97–1.12)
Maliszewska et al. [26]	1.87 (1.53–2.27)	0.69 (0.57–0.82)	0.79 (0.67–0.92)	0.84 (0.72–0.99)	0.87 (0.75–1.01)
Marin-Morales et al. [27]	NR	−0.104 (Beta coefficient)	NR	NR	NR
Maliszewska et al. [28]	1.64 (1.34–2.01)	0.76 (0.58–0.87)	0.78 (0.66–0.93)	NR	NR
Tian et al. [29]	1.12 (1.09–1.21)	NR	NR	NR	NR
Farzaneh et al. [30]	0.368 (Beta coefficient)	−0.171 (Beta coefficient)	−0.162 (Beta coefficient)	NR	NR
Roman et al. [31]	0.01 (0.03–0.10)	−0.03 (0.02–0.04)	−0.05 (−0.06–0.007)	−0.01(0.02–0.005)	−0.02(−0.02–0.06)

NR—Not Reported; Data reported as Odds ratio and 95% Confidence Interval, unless indicated differently.

## Data Availability

Not applicable.
